# MRI-based artificial intelligence to predict infection following total hip arthroplasty failure

**DOI:** 10.1007/s11547-023-01608-7

**Published:** 2023-02-14

**Authors:** Domenico Albano, Salvatore Gitto, Carmelo Messina, Francesca Serpi, Christian Salvatore, Isabella Castiglioni, Luigi Zagra, Elena De Vecchi, Luca Maria Sconfienza

**Affiliations:** 1 Unità Operativa Di Radiologia Diagnostica E Interventistica, IRCCS Istituto Ortopedico Galeazzi, 20161 Milan, Italy; 2 Dipartimento Di Scienze Biomediche Per La Salute, Università Degli Studi Di Milano, 20133 Milan, Italy; 3 DeepTrace Technologies S.R.L., Milan, Italy; 4 Department of Science, Technology and Society, University School for Advanced Studies IUSS Pavia, Pavia, Italy; 5 Department of Physics, Università Degli Studi Di Milano-Bicocca, 20126 Milan, Italy; 6 Institute of Biomedical Imaging and Physiology, Consiglio Nazionale Delle Ricerche, 20090 Segrate, Italy; 7 Hip Department, IRCCS Istituto Ortopedico Galeazzi, 20161 Milan, Italy; 8 Laboratory of Clinical Chemistry and Microbiology, IRCCS Istituto Ortopedico Galeazzi, 20161 Milan, Italy

**Keywords:** Artificial intelligence, Machine learning, Magnetic resonance imaging, Total hip arthroplasty, Infection, Bone edema

## Abstract

**Purpose:**

To investigate whether artificial intelligence (AI) can differentiate septic from non-septic total hip arthroplasty (THA) failure based on preoperative MRI features.

**Materials and methods:**

We included 173 patients (98 females, age: 67 ± 12 years) subjected to first-time THA revision surgery after preoperative pelvis MRI. We divided the patients into a training/validation/internal testing cohort (n = 117) and a temporally independent external-testing cohort (n = 56). MRI features were used to train, validate and test a machine learning algorithm based on support vector machine (SVM) to predict THA infection on the training-internal validation cohort with a nested fivefold validation approach. Machine learning performance was evaluated on independent data from the external-testing cohort.

**Results:**

MRI features were significantly more frequently observed in THA infection (*P* < 0.001), except bone destruction, periarticular soft-tissue mass, and fibrous membrane (*P* > 0.005). Considering all MRI features in the training/validation/internal-testing cohort, SVM classifier reached 92% sensitivity, 62% specificity, 79% PPV, 83% NPV, 82% accuracy, and 81% AUC in predicting THA infection, with bone edema, extracapsular edema, and synovitis having been the best predictors. After being tested on the external-testing cohort, the classifier showed 92% sensitivity, 79% specificity, 89% PPV, 83% NPV, 88% accuracy, and 89% AUC in predicting THA infection. SVM classifier showed 81% sensitivity, 76% specificity, 66% PPV, 88% NPV, 80% accuracy, and 74% AUC in predicting THA infection in the training/validation/internal-testing cohort based on the only presence of periprosthetic bone marrow edema on MRI, while it showed 68% sensitivity, 89% specificity, 93% PPV, 60% NPV, 75% accuracy, and 79% AUC in the external-testing cohort.

**Conclusion:**

AI using SVM classifier showed promising results in predicting THA infection based on MRI features. This model might support radiologists in identifying THA infection.

## Introduction

Hip osteoarthritis is a frequent cause of hip pain and total hip arthroplasty (THA), which, to date, is one of the most common surgical procedures in orthopedic surgery, with the number of implants growing over time due to population aging. THA failure may occur due to prosthetic joint infection and non-septic reasons, such as aseptic loosening, dislocation, adverse reaction to metal debris (ARMD), bone fracture, or implant rupture [1]. Several diagnostic tools exist, including clinical tests, laboratory exams on blood samples and joint fluid, and imaging examinations [2–6]. These tests have some limitations, particularly in detecting THA infection and the responsible microorganism, but all of them are part of a comprehensive preoperative evaluation routinely performed in patients with THA failure. As a matter of fact, there is no highly sensitive and specific single diagnostic preoperative test [7]. Some papers have been published recently on the use of magnetic resonance imaging (MRI) in differentiating septic from non-septic THA failure [8–11]. These studies have investigated the diagnostic performance of different conventional imaging features (e.g., bone edema, synovitis, collections, and bone destruction) and of loco-regional lymphadenopathies reporting very good results. Nevertheless, the interpretation of MRI findings in patients with hip THA can be challenging for radiologists without strong experience in musculoskeletal imaging and other physicians, because these examinations are occasionally performed, generally in referral centers, but also due to artifacts related to the prosthesis itself. In this regard, as in several other settings, artificial intelligence (AI) and machine learning may be supportive to radiologists [12, 13]. AI has several potential applications in augmenting the musculoskeletal radiologist in the assessment of orthopedic implants. Among them, characterization of prosthesis, identification of specific implant models, and assessment of prosthetic positioning and complications. Most works have been published about the use of AI-based analysis of radiographic images to automate postoperative evaluations of joint arthroplasty [14, 15]. For instance, AI-based algorithms reported good accuracy in predicting the dislocation risk of THA (AUC 76.67) or in detecting THA loosening (accuracy 88.3%) [14]. Unfortunately, most of these studies have critical methodological limitations. Thus, current evidence is not sufficient to support the use of AI-based diagnostic algorithms applied to medical images for the evaluation of THA complications in daily clinical practice. The aim of our study was to investigate whether AI can assist radiologists with MRI diagnosis of THA infection.

## Methods

Institutional Review Board of Ospedale San Raffaele, Milano, Italy, approved this retrospective study and waived the need for informed consent (Protocol RETRORAD). After matching imaging, laboratory, and surgical data, our database was completely anonymized to delete any connections between data and patients’ identity according to the General Data Protection Regulation for Research Hospitals.

### Patients

This study was concerned with the assessment of MRI examinations performed by a consecutive series of patients managed at IRCCS Istituto Ortopedico Galeazzi, Milan, Italy, from January 2015 to January 2022. These patients were affected by painful THA requiring revision surgery and were all subjected to preoperative pelvis MRI. We gathered all imaging, surgical, and clinical data of these patients cross-referencing the database of our Radiology Department with that of our Hip Department and Laboratory of Clinical Chemistry and Microbiology. The following inclusion criteria were considered: (i) patients subjected to first-time revision procedures for monolateral failed THA; (ii) availability of intraoperative microbiological tests; and (iii) preoperative unenhanced pelvis MRI performed up to 1 month before revision surgery with the same imaging protocol. The exclusion criteria were: (i) nondiagnostic MRI due to prosthesis-related artifacts; (ii) inflammatory arthritis, tumors, bowel inflammatory disease, or autoimmune disease. Overall, a total of 173 patients were included in this study (98 females, 75 males; mean age: 67 ± 12 years, range 28–94). They were divided into training/validation/internal-testing and external-testing cohorts as detailed below in the machine learning section.

### Microbiologic analysis

Intraoperative samples from periprosthetic material collected during revision surgery (membranes, bones, fluid, THA components) were sent to our laboratory for culture analysis. Agar plates and enrichment broths were incubated for 48 h and 15 days, respectively, and daily checked for microbial growth. In case of broth turbidity, an aliquot was plated onto blood agar. Colonies grown from agar plates were identified by biochemical testing performed on a Vitek 2 analyzer (BioMerieux, Marcy L’Etoile, France). The final diagnosis of THA infection was obtained according to the International Consensus Meeting Criteria [16], which include two or more samples of intraoperative cultures showing the same microorganism growth and preoperative blood test results concerning the levels of C-reactive protein (CRP), erythrocyte sedimentation rate (ESR), and synovial fluid analysis that were used, together with clinical data, imaging findings, culture analysis from preoperative joint aspiration and intraoperative samples, to reach the final diagnosis.

### MRI protocol and images interpretation

All MRI scans were done at in the same1.5 T unit (Avanto, Siemens Medical Solutions, Erlangen, Germany) at our institution. A combination of the table-integrated coil and abdominal coil was used. Our metal artifact reduction sequence (MARS) protocol included coronal T1-weighted, coronal STIR, axial T1-weighted, axial T2-weighted, and sagittal T2-weighted images [17]: coronal T1-weighted (repetition time/echo time, 500/9.1 ms; number of excitations, 2; slice thickness, 3 mm; turbo factor, 6; flip angle, 180; voxel size, 1.3 × 0.9 × 3 mm; bandwidth, 302), coronal STIR (5180/81 ms; number of excitations, 2; slice thickness, 3 mm; inversion time, 160 ms; turbo factor, 28; flip angle, 122; voxel size, 1.4 × 1.1 × 3 mm; bandwidth, 395), axial T2-weighted (5000/68 ms; number of excitations, 2; slice thickness, 3.2 mm; turbo factor, 27; flip angle, 150; voxel size, 1.4 × 1.0 × 3.2 mm; bandwidth, 383), axial T1-weighted (593/9.1 ms; number of excitations, 3; slice thickness, 3.2 mm; turbo factor, 6; flip angle, 180; voxel size, 1.2 × 0.9 × 3.2 mm; bandwidth, 302), sagittal T2-weighted (3550/85 ms; number of excitations, 2; slice thickness, 3.5 mm; turbo factor, 26; flip angle, 150; voxel size, 1.0 × 1.0 × 3.3 mm; bandwidth, 381). The field of view was adapted to patient’s body build including the whole pelvis and all prostheses. All MRI scans were reviewed by a radiologist (blinded to clinical and microbiologic data) with 9 years of experience in musculoskeletal imaging, who provided a yes/no answer for each MRI feature. As done in a previous study on pelvis MRI in patients with THA [8], MRI features of THA failure were: periprosthetic bone destruction, periprosthetic soft-tissue mass, effusion, synovitis, lamellated synovitis, extracapsular edema, fibrous periprosthetic membrane, bone edema, and extracapsular collection/sinus tract.

### Machine learning analysis

The machine learning analysis was performed using the TRACE4© platform (DeepTrace Technologies, Milan, Italy) [18].Our population of study was divided into training/validation/internal-testing and external-testing cohorts based on MRI performed before and after September 2019, respectively. The training/validation/internal-testing cohort consisted of 117 patients (38 with THA infection and 79 without THA infection). The external-testing cohort consisted of 56 patients (19 with THA infection and 37 without THA infection). All imaging parameters previously assessed by the musculoskeletal radiologist were used to train, validate and test the machine learning algorithm based on support vector machine (SVM, with Gaussian kernel) to predict THA infection on the training/validation/internal-testing cohort. A nested fivefold validation (10 ensembles, 250 trained models) approach was employed. Thereafter, machine learning performance was evaluated on temporally independent data from the external-testing cohort.

### Statistical analysis

Chi-square statistics were used to compare each MRI feature between patients with infected and non-infected THA. Sensitivity, specificity, positive predictive value (PPV), negative predictive value (NPV), accuracy, area under the curve (AUC), and odds ratios (OR) were calculated for each MRI feature. We also assessed the diagnostic performance of a combination of conventional MRI features with the highest OR. Continuous variables were reported as mean ± standard deviation. Discrete variables were summarized as median and interquartile range. Bonferroni correction for multiple comparisons was applied and statistical significance was set at *P* < 0.003. SPSS software (v. 26, IBM, Armonk, NY) was used for statistical analysis. AUC, accuracy, sensitivity, specificity, PPV, and NPV of the machine learning classifier were calculated in the training-internal validation and test cohorts, respectively.

## Results

**Fig. 1:**
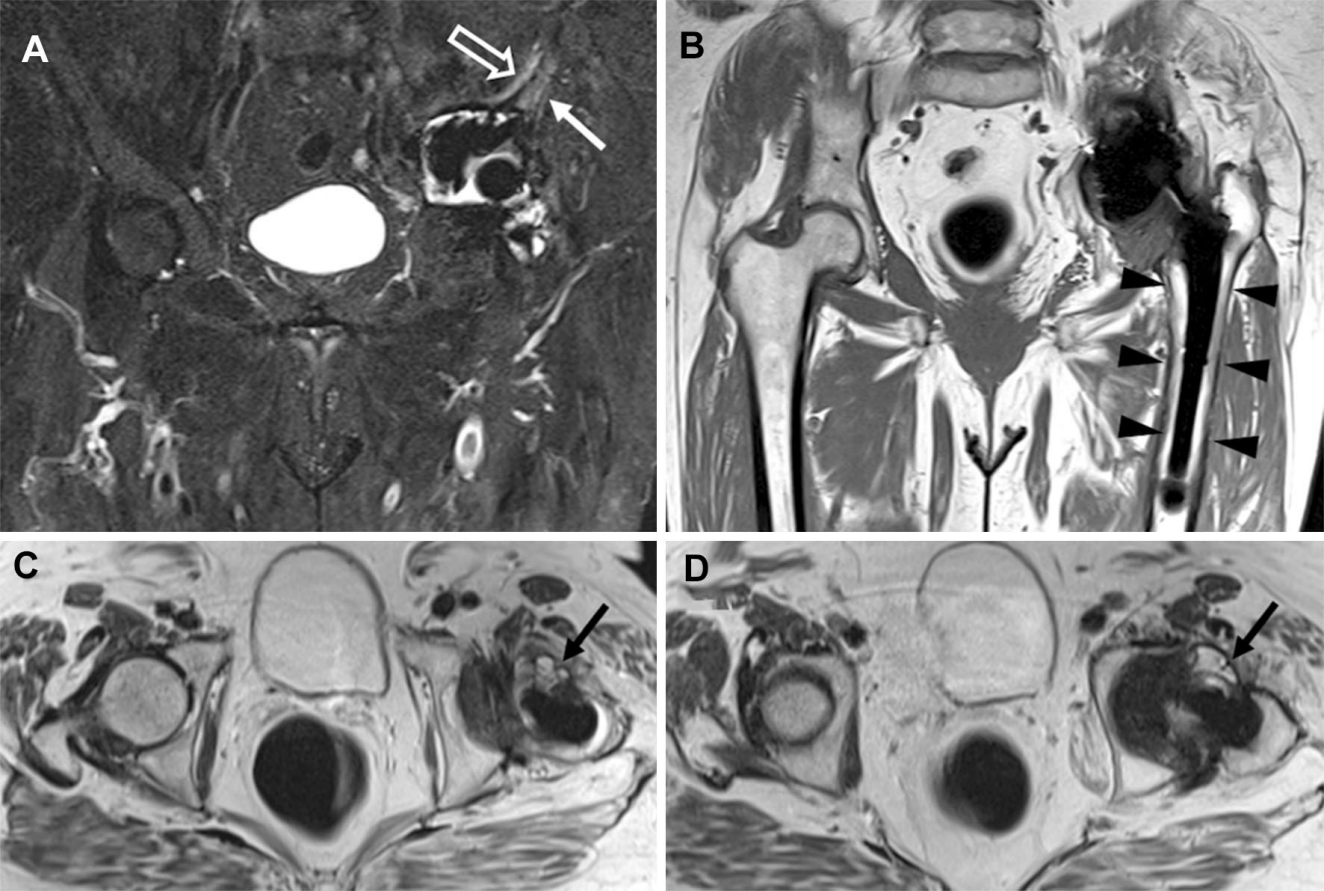
Pelvis MRI of a 77-year-old female with THA infection. Coronal STIR (**A**) shows periprosthetic acetabular bone edema (white arrow) and extracapsular edema (void arrow). No bone destruction is noted in the coronal T1-weighted image (**B**). Axial T2-weighted images (C and D) show effusion and synovitis

Conventional MRI features were significantly more frequently observed in patients with THA infection compared to patients without infection (all with *P* < 0.001), except bone destruction (*P* = 0.155), periarticular soft-tissue mass (*P* = 0.005), and fibrous membrane (*P* = 0.081). The values of sensitivity for detecting THA infection ranged from 5.4% (periarticular soft-tissue mass) to 80.7% (bone edema), specificity from 51.7% (bone destruction) to 97.4% (lamellated synovitis), PPV from 10.3% (periarticular soft-tissue mass) to 87.9% (synovitis), NPV from 62.5% (periarticular soft-tissue mass) to 89% (bone edema), accuracy from 46.8% (bone destruction) to 81.5% (synovitis), and OR from 0.198 (periarticular soft tissue mass) to 29 (synovitis). Data on diagnostic performance of all MRI features are reported in Table 1. Figure 1 shows a representative case from our study population.

**Table 1:** Diagnostic performance of all MRI features in detecting infected THA

	Bone destruction	Periarticular mass	Effusion	Synovitis	Lamellated synovitis	Extracapsular edema	Fibrous membrane	Bone edema	Extracapsular collections
Sens	36.8%	5.4%	73.7%	50.9%	29.8%	64.9%	36.8%	80.7%	50.9%
Spec	51.7%	77.8%	59.5%	96.6%	97.4%	87.1%	75.9%	76.7%	81.0%
PPV	27.3%	10.3%	47.2%	87.9%	85.0%	71.2%	42.9%	63.0%	56,9%
NPV	62.5%	63.2%	82.1%	80.0%	73.9%	83.5%	71.0%	89.0%	77.0%
Acc	46.8%	54.3%	64.2%	81.5%	75.1%	79.8%	63.0%	78.0%	71.1%
OR	0.625	0.198	4.111	29.000	16.008	12.457	1.833	13.785	4.425
P	0.155	0.005	< 0.001	< 0.001	< 0.001	< 0.001	0.081	< 0.001	< 0.001

Sens.: sensitivity; Spec.: specificity; PPV: positive predictive value; NPV: negative predictive value; Acc.: accuracy; OR: odds ratios; *P*: *P*-value with statistical significance set at *P* < 0.003

When all MRI features were considered in the training/validation/internal-testing cohort, SVM classifier reached 92% sensitivity, 62% specificity, 79% PPV, 83% NPV, 82% accuracy, and 81% AUC in predicting THA infection, with bone edema, extracapsular edema, and synovitis resulting the best predictors ranked by descending importance. After being tested on the external-testing cohort, the classifier showed 92% sensitivity, 79% specificity, 89% PPV, 83% NPV, 88% accuracy, and 89% AUC in predicting THA infection.

SVM classifier showed 81% sensitivity, 76% specificity, 66% PPV, 88% NPV, 80% accuracy, and 74% AUC in predicting THA infection in the training/validation/internal-testing cohort based on the only presence of periprosthetic bone marrow edema on MRI, while it showed 68% sensitivity, 89% specificity, 93% PPV, 60% NPV, 75% accuracy, and 79% AUC in the external-testing cohort.

## Discussion

The main finding of our study is the validation of a diagnostic machine learning classifier to support physicians in the interpretation of imaging findings when dealing with preoperative MRI of patients with failed THA.

Few papers have investigated the diagnostic performance of preoperative MRI in predicting infection in patients with THA infection of the hip [8–10, 19] and of the knee [20, 21]. Our results, in line with previous studies, highlight the accuracy of some MRI features in predicting THA infection, such as bone edema, synovitis, synovial layering, and extracapsular edema with accuracy of 75.1%–81.5%. In more detail, the most specific findings were synovitis, lamellated synovitis, and extracapsular edema (specificity of 96.6%, 97.4%, and 87.1%, respectively), while bone edema reached the highest values of sensitivity (80.7%) and NPV (89%). On the other hand, bone destruction, periarticular soft tissue, and periprosthetic fibrous membrane are imaging findings occasionally observed in THA infection, being more commonly seen in aseptic THA failure. According to these data, the AI-based SVM classified bone edema, extracapsular edema, and synovitis as the best predictors of THA infection. Based on all MRI features, the classifier showed 82% accuracy, but with remarkable unbalance between sensitivity (92%) and specificity (62%). However, a substantial increase of specificity (79%), PPV (89%), accuracy (88%), and AUC (from 81 to 89%) was observed in the external-testing cohort. Bone edema was selected by the AI-based SVM as the best predictor showing 80% accuracy in the training/validation/internal-testing cohort and 75% accuracy in the external-testing cohort. Again, a substantial improvement in specificity (from 76 to 89%) and PPV (from 66 to 89%) was observed in the external-testing cohort. These results demonstrate that AI-based machine learning classifiers might be useful supportive diagnostic tools in challenging conditions like the differentiation of septic and aseptic THA failure. Indeed, AI tools applied to imaging after arthroplasty may improve reporting activity and decrease the mistakes rate by reducing cognitive load and fatigue for radiologists [15]. Unfortunately, a comparison with previously published data is not possible, since, to our knowledge, no other studies investigated the diagnostic performance of an AI-based machine learning classifier built upon MRI features in this setting. Moreover, while AI models have been proven for detecting implant complications such as dislocations and loosening on radiographs [22], poor attention has been placed on the tremendous clinical impact that might have AI-based supportive tools in the diagnosis of THA infection.

Some limitations of our study must be pointed out. First, the relatively limited number of patients included in our series. It is well known that a huge amount of data is essential to build robust machine learning classifiers [23–26]; thus, larger studies may prove even higher diagnostic performance of AI-based predicting models in failed THA. It should be noted that collecting preoperative pelvis MRI in patients with failed THA eligible for revision surgery is not so easy. Then, we cannot exclude that our evaluation could have been affected by susceptibility artifacts, despite the use of metal artifact reduction sequences, particularly concerning some imaging features of the periprosthetic bone (bone edema, bone destruction, fibrous membrane), that may be missed or wrongly seen. New technologies included in the most recent MRI scanners reduce the risk of misinterpretation of images and may allow improving the diagnostic performance of both radiologists and AI-based supportive classifiers. Last, we did not include in our analysis the evaluation of locoregional lymphadenopathies that some authors have recently reported as potential imaging biomarkers of infected THA, even with higher diagnostic performance reported by conventional MRI features [8, 27].

In conclusion, AI using SVM classifier showed promising results in predicting THA infection based on MRI features assessed preoperatively. This model might represent an adjunctive tool to support radiologists in identifying THA infection and could form the basis for further trials in this little explored field. Future studies are warranted to build AI-based models that combine imaging, clinical, and laboratory data to improve the accuracy of preoperative evaluation of patients undergoing revision surgery for failed arthroplasty.

## Declaration

### Ethcial approval

The study was conducted in accordance with the Declaration of Helsinki,and approved by the Institutional Review Board of Ospedale San Raffaele, Milano, Italy (Protocol name: RETRORAD, approved by Comitato Etico OSR).
